# Comparability between AI and human cognition and its role in psychological research and AI ethics

**DOI:** 10.1111/bjop.70056

**Published:** 2026-01-18

**Authors:** Janet H. Hsiao

**Affiliations:** ^1^ Division of Social Science Hong Kong University of Science and Technology Hong Kong; ^2^ Department of Computer Science & Engineering Hong Kong University of Science and Technology Hong Kong

**Keywords:** computational modelling, ethical AI, explainable AI

## Abstract

With the advances in AI technology, comparison studies between humans and AI can not only enhance our understanding of information processing mechanisms underlying human cognition but also facilitate our understanding of AI systems' behaviour and interactions with humans. In particular, explainable AI (XAI) methods, including both computational and experimental methods, can be used to reveal the mechanisms underlying AI's behaviour and its interactions with humans. This information can be used (1) as computational models to study human behaviour, (2) for updating users' beliefs about AI during the interactions, and (3) for evaluation purposes to examine potential ethical issues associated with AI adoption. Different AI systems may require different XAI methods to accurately reveal their underlying mechanisms to facilitate the comparisons with humans. Thus, an important future research direction is to develop task‐specific XAI methods through interdisciplinary approaches across psychology and AI to benefit both psychological research and the development of ethical AI.

In psychological research, computational modelling has been an important method for understanding human cognition in terms of its representational and computational mechanisms through comparisons between humans and computational systems (Hsiao, 2024). Such comparisons also facilitate our understanding of computational or artificial intelligence (AI) systems' behaviour and interactions with humans. In particular, the adoption of AI technologies in society often comes with some ethical concerns, especially in critical public systems. In addition to issues on accuracy and robustness, there is a high demand on explainability to ensure AI's behaviour is consistent with human expectations. A key issue has been how to help the stakeholders develop a robust mental model of AI, which can in turn induce an appropriate level of trust and ensure ethical AI adoption. More specifically, humans engage an important cognitive capacity, Theory of Mind (ToM), to infer others' mental states and behaviour during social interaction. When AI systems demonstrate emergent intelligent behaviour similar to humans, their users may attribute mental states to them through the ToM capacity and assume that they possess similar social and cognitive skills and would behave similar to regular humans. This assumption may lead to a false sense of security, inappropriate level of trust and unsafe AI adoption. This risk can be mitigated if the users update this belief through learning about how these systems differ from regular humans (Yang et al., 2021, 2022). Thus, research on the comparability between AI and human cognition, or more specifically similarities and differences between AI and human cognition in internal information processing mechanisms and exhibited behaviour, plays an essential role not only for psychological research but also for enhancing AI explainability and safe adoption in society (Hsiao, 2024; Hsiao & Chan, 2023).

In this special issue on the use of AI in psychological research, Sano et al. (2024) compared attended facial features by humans vs. a deep‐learning model in facial impression judgements. They found that humans typically attended to the triangular region formed by the two eyes and the nose, consistent with previous studies of face perception. In contrast, visual features contributed to the deep‐learning model's impression judgements derived using the explainable AI (XAI) method Grad‐CAM (gradient‐based) (Selvaraju et al., 2020) and covered a broader area, including parts of the forehead and the chin. They argued that this difference may be because human visual attention is driven by both stimulus salience and task‐relevant information, whereas the model's attended features are mainly task‐driven. Accordingly, by comparing the attended features in humans and in models, we may infer what aspects of human attention could be task‐driven or mainly stimulus‐driven.

Similarly, Qi, Zheng, et al. (2024) showed that in object recognition, whereas humans only attended to the most diagnostic features for recognition, deep‐learning models (ResNet50; He et al., 2016) used all features relevant to the identification of the object class in parallel. They pointed out that in contrast to AI models where all features can be processed in parallel, humans process different aspects of visual information one at a time through a series of eye fixations. Thus, humans optimize the processing by attending to areas with the most diagnostic features for the task to more efficiently accumulate perceptual evidence for the recognition decision. Interestingly, Qi, Zheng, et al. (2024) also reported individual differences in human attention strategies during object recognition (focused vs. explorative scanning), and that the model's strategy aligned better with those adopting the explorative scanning strategy. This suggests that individual differences may be an important factor to consider when we evaluate AI systems' alignment with human cognition for providing explanations or for benchmarking purposes (see also Qi et al., 2025; Yang et al., 2023).

Note that Qi, Zheng, et al. (2024) also compared different XAI methods for revealing features contributing to the AI model's output and found that feature maps generated by perturbation‐based methods (pixel‐coverage‐corrected RISE; Petsiuk et al., 2018; Xie et al., 2022) had higher similarity to human attention maps than those generated by gradient‐based methods such as Grad‐CAM. Indeed, different visualization XAI methods may result in slightly different feature maps. A common way to evaluate the quality of an XAI feature map is to calculate its faithfulness, that is, how accurately the XAI feature map highlights input features that can lead to changes in AI's output (Samek et al., 2017). Different AI models may require different XAI methods to accurately capture their information use for decision‐making and achieve high faithfulness (see, e.g., Liu et al., 2024, and Zhao et al., 2024, for object detection). When comparing XAI feature maps with corresponding human data, it is essential to first confirm whether the XAI feature maps generated have achieved a good faithfulness.

Issues related to AI systems' explainability were also raised in Mueller et al. (2024), where the authors examined whether knowing about the decisions of an automated face recognition (AFR) system with high accuracy could enhance humans' face recognition performance. Interestingly, they found that even though participants were told about the AFT system's high accuracy, they regularly overruled the system's decisions, resulting in limited performance improvement. This phenomenon is consistent with previous research suggesting humans' tendency to compare AI's information processing with their own when making decisions (Hsiao & Chan, 2023; Yang et al., 2021, 2022). In addition, when participants were given only the AFR system's binary match/mismatch decisions, they showed a shift towards making match responses and became overconfident in difficult trials. Importantly, providing information about the AFR system's face similarity scores could mitigate these biases. This finding demonstrated the importance of explanations, or more specifically information about AI's internal mechanisms, for mitigating potential biases or unintended use due to misunderstanding.

Sometimes ethical issues related to AI adoption may not be readily identifiable, and explicit evaluations are often required. Percell et al. (2024) reported one such case involving self‐other differences in humans' attitudes towards AI‐mediated communication, where participants expected others to use these tools more often than themselves and perceived it as more acceptable than their own use. This finding has important ethical implications regarding what usage should be considered acceptable or unacceptable to ensure fairness and accountability and minimize potential risks such as misinformation for AI governance. In another study, Bilalic et al. (2024) found that after the recent AI chess breakthrough related to the advances in deep learning methods in the 2010s, we have not yet observed a corresponding performance gain in human chess players. This is in contrast to the previous AI chess breakthrough in the 1990s where young top players demonstrated a marked accelerated performance gain, benefiting from the accessibility to advanced AI chess systems to accumulate chess knowledge. This phenomenon could be related to the low explainability of these deep‐learning AI chess systems (see Hammersborg & Strümke, 2024, for XAI methods for these models). Indeed, different AI applications may be associated with different issues related to their use, which will require different explanation and evaluation methods to identify or mitigate (see, e.g., Liu et al., 2024 and Qi, Liu, et al., 2024, for computer vision models, and Qi et al., 2025 and Zhao et al., 2025, for large language models or transformer models). An important research direction thus is to develop a comprehensive taxonomy of AI risks and ethical issues across AI applications and the associated evaluation and explanation methods, where psychological studies and research methods play an essential role (e.g., artificial cognition; see Taylor & Taylor, 2021).

In summary, this special issue has illustrated the importance of research on comparability between AI and human cognition in both psychological research and the development of ethical AI systems. On the one hand, computational models simulating the human mind have provided powerful methods to enhance our understanding of its complexity (see also Cohen & Quinlan, 2025; Guo et al., 2025; He et al., 2024; Jones et al., 2024; Phillips & White, 2025). On the other hand, methods developed in psychological research have enabled us to examine AI systems' behaviour and internal mechanisms, comparability to human cognition, and interactions with human users, facilitating the identification of potential ethical issues and the development of mitigation strategies (see also Laricheva et al., 2024; Xu & Wang, 2024). Currently, there is still limited research on XAI methods for different AI applications to facilitate comparison studies between AI and humans. Looking ahead, adopting a collaborative approach to integrate the diverse perspectives and expertise across psychology and AI appears to be essential for developing novel methods to enhance the understanding of the nature of both human cognition and artificial cognition, as well as their interaction.

## AUTHOR CONTRIBUTIONS

**Janet H. Hsiao:** Conceptualization; writing – original draft; writing – review and editing.

## Data Availability

No dataset was generated in this commentary and thus data sharing is not applicable.
